# Research status and prospects of acupuncture for autism spectrum disorders

**DOI:** 10.3389/fpsyt.2023.942069

**Published:** 2023-05-25

**Authors:** Xiang Li, Ji-Cheng Li, Qi-Qi Lu, Fan Zhang, Shan-Qiang Zhang

**Affiliations:** ^1^Clinical Research Institute, Zhejiang Provincial People’s Hospital, Hangzhou Medical College, Hangzhou, China; ^2^Department of Anatomy, Shantou University Medical College, Shantou, China; ^3^Medical Research Center, Yuebei People’s Hospital, Shantou University Medical College, Shaoguan, China; ^4^Institute of Cell Biology, Zhejiang University School of Medicine, Hangzhou, China

**Keywords:** autism spectrum disorder, traditional Chinese medicine, clinical trial, acupuncture, meridian

## Abstract

Autism spectrum disorder (ASD) is a neurodevelopmental disorder and has a predilection for children. Its symptoms, such as lifelong social communication deficits and repetitive sensory-motor behaviors, put a huge burden on the patient’s family and society. Currently, there is no cure for ASD, and some medications that can improve its symptoms are often accompanied by adverse effects. Among many complementary and alternative medicine (CAM) therapies, acupuncture has shown promising application potential, but after years of practice, it has not been recognized as the preferred CAM therapy for ASD. Therefore, we analyzed and discussed the clinical study reports of acupuncture in the treatment of ASD in the past 15 years from the aspects of study subjects, group setting, intervention modalities, acupoint selection, outcome evaluation, and safety. The data accumulated at present are not sufficient to support the clinical effectiveness of acupuncture in ASD and to justify its use in clinical practice. They provide, however, initial evidence of possible effectiveness and encourage further investigation in order to reach firm conclusions. Based on a comprehensive analysis, we believed that following the Standards for Reporting Interventions in Clinical Trials of Acupuncture (STRICTA) and Consolidated Standards of Reporting Trials (CONSORT), screening the optimal combination of acupoints applying a rigorous scientific study design, and performing the related functional experiments may be the effective way to convincingly test the hypothesis that acupuncture may be beneficial in ASD patients. The significance of this review is to provide a reference for researchers to carry out high-quality clinical trials of acupuncture in the treatment of ASD from the perspective of the combination of modern medicine and traditional Chinese medicine.

## 1. Introduction

Autism spectrum disorder (ASD), also known as autism, is a kind of neurodevelopmental disorder characterized by communication deficits, social impairments, repetitive and stereotyped behaviors, and sensory-motor coordination defects (1). The latest diagnostic systems, the International Classification of Diseases 11th Revision (ICD-11) and Diagnostic and Statistical Manual of Mental Disorders, Fifth Edition, revised (DSM-5-TR) used the term ASD to summarize autism, Asperger syndrome, and several other diseases (2). The World Health Organization estimated that one in 160 children globally has ASD, with a male-to-female ratio of 4:1 (3). The number of reported ASD cases has increased approximately 20-fold over the past 30 years. Currently, the prevalence of ASD in the United States and China has exceeded 2%, of which the number of patients in China has exceeded 10 million, increasing at a rate of nearly 200, 000 cases every year (4, 5). ASD is child-specific, and its symptoms mostly manifest in early childhood and persists throughout life, which has a serious negative impact on the patients’ daily life, family, and social development. Therefore, active prevention and treatment of ASD has always been an important work committed by medical workers all over the world.

At present, the pathogenesis of ASD remains undefined, but some researchers believed that genetic, environmental, neurodevelopmental, and immune factors play an important role in its occurrence and development (1, 2). There has been no effective treatment method for ASD due to the lack of sufficient evidence to clarify its mechanism (3). In modern medicine, several drugs have been used to treat ASD, but these drugs are only used to target specific symptoms or comorbidities rather than ASD (2, 3). Although risperidone and aripiprazole can improve patients’ agitation and irritability, they are often accompanied by adverse effects such as weight gain, nausea, sedation, and hyperglycemia (2). In addition, methylphenidate, atomoxetine, and guanfacine have been used to improve attention-deficit and hyperactivity disorder, but can cause adverse effects such as sleep disruption, loss of appetite, nausea, irritability, fatigue, sedation, and hypotension (2, 6). In addition to pharmacological therapy, behavioral and educational intervention (BEI) has also been used in the treatment of ASD, but most high-quality BEI need 20–40 h of treatment per week, and it takes a long time to show benefits (7, 8). Therefore, both doctors and patients’ families are actively searching for an effective alternative or complementary therapy for ASD (9).

Complementary and alternative medicine (CAM) was first proposed by the National Institutes of Health (NIH) in the United States. It refers to medical care practice outside of modern mainstream medicine. Complementary medicine refers to the diagnosis and treatment methods used simultaneously with alternative conventional medicine. Alternative medicine refers to the method of treating diseases instead of modern mainstream medicine (6, 10, 11). The regimens of CAM have been tried in 52–74% of ASD patients, and even some patients received at least seven CAM therapies (10–12). Currently, the CAM therapies for ASD can be divided into biologically based treatments and non-biologically based treatments (13). Biologically based treatments include dietary interventions, nutraceuticals, hyperbaric oxygen therapy, and chelation. Although studies have found that eliminating gluten and casein in diet is beneficial to ASD patients, such trials are often difficult to be effectively controlled due to the lack of control group, poor diagnostic characteristics, small sample size, and non-standardized outcome measurement (14, 15). Chelation has various adverse effects, especially the loss of essential elements in the body during the treatment process (16). The National Center for Complementary and Alternative Medicine (NCCAM) has divided non-biologically based treatments into three groups: mind–body medicine (including yoga, music, and dance), manipulative and body-based practices (such as massage, chiropractic care, and acupuncture), and energy medicine (such as Reiki or homeopathy) (13). To date, all these therapies have been observed to be beneficial in treating ASD to some extent, but there is not enough evidence to confirm their clinical effectiveness (17). Acupuncture has been shown to be more promising in the treatment of ASD (18).

Acupuncture is a unique treatment method of traditional Chinese medicine (TCM). It consists of inserting the needle into the specific acupoints of the patient’s body at a certain angle, and then the acupoint is stimulated with twirling, lifting, and thrusting of the needle, so as to achieve the purpose of treating diseases (19, 20). Acupuncturist usually take the subject’s feeling of “*De-qi*”(气, 针感) as the standard to judge the treatment response (21). In China, acupuncture has been practiced for thousands of years. Many ancient TCM books have recorded its efficacy in treating a variety of diseases, including neurological and mental diseases, such as stroke, depression, and Alzheimer’s disease (22). At present, acupuncture has spread to many countries and regions around the world, especially in the United States (23). It is reported that 22 states in the United States licensed acupuncturists through the Medical Board, and 48 states have statutes and regulations that determine licensure and medical professional training requirements (24). However, despite this development, acupuncture has not been recognized as the authoritative CAM recommendation for the treatment of mental diseases due to the lack of sufficient scientific evidence, which is why we carried out this review (25).

Unlike modern medicine, which only aims at the disease itself or symptoms, the principle of TCM treatment of diseases emphasizes the overall concept and syndrome differentiation (23). The advantage of this medical concept is that it can treat patients individually and reduce the probability of adverse events (22). In TCM, ASD has no corresponding term, but its symptoms are in line with the category of “*Shen zhi*” diseases (神志病; mental disorders), such as “*Dian zheng*” (癫证) (sluggishness, depression, irritability, and decreased appetite), “*Wu chi zheng*” (五迟证) (delays in standing, walking, hair, teeth, and speech development), and “*Jie lu*”(解颅) (delayed closure of fontanels, delayed bone development, which is similar to congenital or acquired hydrocephalus in modern medicine) (26–29). The pathogenesis of ASD in TCM is related to the congenital deficiency of the *Zang-Fu* (viscera) organs such as the heart, liver, spleen, and kidney, as well as their acquired mutual regulation imbalance (26). Therefore, TCM divides ASD into four syndrome types, namely, syndrome of exuberant fire of heart and liver (心肝火旺证), syndrome of heart spirit confused by phlegm (痰蒙心窍证), deficiency of heart and spleen syndrome (心脾两虚证), and deficient kidney essence syndrome (肾精不足证) (27). In terms of treatment, TCM restores the internal balance of the body by clearing the heart and calming the liver, clearing the heart and purging fire, refreshing the brain and opening the orifices, promoting Yang, and relieving heat (26). Correspondingly, according to the need of different syndromes, acupuncturists will select a group of acupoints in various parts of the human body, such as head (scalp acupuncture), ear (auricular acupuncture), tongue (tongue acupuncture), and trunk and limbs (body acupuncture) for a certain session of treatment (30–32). Compared with other CAM therapies of modern medicine, acupuncture has the advantages of low cost, lasting effect, and no serious side effects (22). With the development of medical technology, acupuncture has also derived a variety of therapies such as laser acupuncture (LA), electroacupuncture (EA), and transcutaneous electrical acupoint stimulation (TEAS), which have been proved to be suitable for the treatment of a variety of human diseases (33, 34). However, because the theory of TCM is complex and difficult to understand, there are differences in the mastery of knowledge among acupuncturists (35). In addition, the clinical application of acupuncture also lacks a universal international authoritative standard. Therefore, the analysis and discussion of previous studies will help to formulate unified standards and carry out relevant high-quality research in the future.

Several articles have analyzed the clinical studies on acupuncture in the treatment of ASD in recent years, but most authors have not made an in-depth interpretation of the relevant TCM knowledge of ASD and the function of acupoints, especially the potential relationship between TCM and acupuncture and modern medicine (3, 17, 36, 37). Therefore, we reviewed the clinical study reports on acupuncture in the treatment of ASD in past 15 years. The reports mainly included randomized, controlled, and double-blinded trial, pilot study, case report, prospective single-blinded controlled study, and retrospective study on manual acupuncture, EA, LA, and TEAS in the treatment of ASD. These reports are analyzed and discussed from the aspects of study subjects, group setting, intervention modalities, acupoints selection, outcome evaluation, and safety. Furthermore, we also discussed the relevant mechanisms of some acupoints selected in these reports by associating TCM with modern medicine in order to provide a more comprehensive reference for the development of acupuncture in the treatment of ASD.

## 2. Clinical trials

The important contents in the clinical study reports of acupuncture in the treatment of ASD are summarized in Table 1. It is worth noting that in order to perform comprehensive analysis and discussion, we did not limit the types of clinical trials. Because there were few reports in English language on clinical studies of acupuncture in the treatment of ASD, additional screening criteria may result in too little literature and limited to a small number of fixed research teams, which may make it difficult for readers to fully understand the progress of this type of research.

**Table 1:** The studies on acupuncture in the treatment of ASD.

Clinical study	Study design	Grouping and number of patients	Therapeutic method	Frequency	Total treatment times	Acupoints		Efficacy evaluation measures	Improvement rate (%)	Adverse effects
						Respective	Frequently used (%)			
Surapaty et al. (38)	Randomized control	LA: 23 Control: 23	LA	3/week	18	GV20, HT7, LI4, SP6, ST36, GB34, LR3	SP6 (45.5), ST36(36.4), GV20 (27.3), LI4 (27.3), HT7 (27.3), EX-HN1 (27.3), EX-HN3(27.3)	WeeFIM^®^, parental-report sensory-profile questionnaire	Expression score: 16/23 (70%) Expression score: 20/23 (87%) Parental-report score: 10/23 (44%)	Non-reported
Lee et al. (6)	Randomized control	H + A: 120 P + A: 120	HM, MA	2/week	24	EX-HN1, GV20, GV24			CARS, ABC^1^, ABC^2^, SMS, CHQ-PF28, EQ-5D-Y Proxy 1, K-PSI-SF	Non-reported	Non-reported
Yau et al. (9)	Pragmatic study	Regression: 21 Natal: 47	MA	2/week	30	GV20, EX-HN3, mid line of forehead, lateral line 2 of forehead, posterior lateral line of vertex, primary auditory cortex, and auditory speech area			Rating scale for quantifying symptoms of ASD based on Blatt-Kupperman index	66/68 (97%)	Non-reported
Warren et al. (39)	Observational pilot study	AA: 10	MA	2/week	8	Non-reported			BASC-2, CRS-R, PDDBI, CSHQ, PSI	9/10 (90%)	Non-reported
Zhao et al. (40)	Before-after study	EA: 55	EA	2/day	720–1,440	LI4, LI11, ST36, SP6,			SPECT	31/55 (56.4%)	Non-reported
Zhang et al. (41)	Prospective single-blinded controlled study	TEAS: 37 Control: 39	TEAS	5/week	60	LI4, PC6, ST36, SP6			EIA, CARS, ABC^2^, ADQ, Sleep Status and Food Choice Questionnaires, parental report, plasma AVP and OXT	emotional response (*p* = 0.037); fear or anxiety (*p* = 0.008); level and consistency of intellective relations (*p* = 0.023); general impressions (*p* = 0.030); total score (*p* = 0.042) of CARS; areas of sensory (*p* = 0.006) and relating (*p* = 0.013); the number of accepted food (*p* = 0.046); The plasma levels of AVP (*p* = 0.007) and OXT (*p* = 0.041)	Non-reported
Wong et al. (42)	Randomized control	EA: 30 SEA: 25	EA	3/week	12	EX-HN1, EX-HN3, PC6, HT7, LR3, AT3, TF4, SP6			WeeFIM®, PEDI, Leiter-R, CGI-I, ABC^2^, RFRLS, RDLS, parental report	language comprehension (*P* = 0.02); self-care assistant domain (*p* = 0.028) of PEDI, and CGI-I (*p* = 0.003); social initiation (*p* = 0.01); receptive language (*p* = 0.006); motor skills (*p* = 0.034); coordination (*p* = 0.07); attention span (*p* = 0.003)	Non-reported
Wong et al. (43)	Randomized control	TA: 25 SA: 25	MA	5/week	40	Run Ze, Guan Zhu, Tian Men, Di You			WeeFIM®, GMDS, RDLS, RFRLS, SPT	Self-Care (Total Score, *p* < 0.0005; Functional Quotient, *p =* 0.007); Cognition (Total Score, *p =* 0.006; Functional Quotient, *p* < 0.0005)	Non-reported
Chan et al. (44)	Randomized control	MA:16 Control: 16	MA	5/week	30	Non-mentioned			qEEG	Language and Social Interaction: 16/16 (100%)	Non-reported
Allam et al. (45)	Randomized control	MA: 10 Control: 10	MA	2/week	50	DU 20, DU 26, GV17, 3 temple needles, Yamamoto YNSA 2 points: cerebrum and aphasia points			language test	cognitive and expressive language skills: 10/10 (100%)	Non-reported
Chen et al. (46)	Case study	Acupoint A: 1 Acupoint B: 1	EA	3/week	24	Acupoint A set: EX-HN1, EX-HN3, PC6, AT3, ST36, LR3 Acupoint B set: EX-HN1, EX-HN3, HT7, Shounaodian, KI3, SP6			ABC^2^, RFRLS, WeeFIM®, CGI-I, parental report	Irritability and Stereotypy: 1/2 (50%); sensory motor and response: 2/2 (100%); affectual response: 1/2 (50%); social relatedness, Communication, and Stereotypy behavior: 1/2 (50%)	Non-reported

H + A, herbs + acupuncture; P + A, placebo + acupuncture; HM, herbal medicine; MA, manual acupuncture; CARS, Childhood Autism Rating Scale; ABC^1^, Autism Behavior Checklist; ABC^2^, aberrant behavior checklist; CHQ-PF28, Child Health Questionnaire-parent Report Form 28; EQ-5D-Y Proxy 1, EuroQoL Five-dimension Five-level Youth proxy 1; K-PSI-SF, Korean Parenting Stress Index Short Form; LA, laser acupuncture; WeeFIM, The Functional Independence Measure for Children; ASD, autism spectrum disorders; AA, acupressure and acupuncture; BASC-2, Behavior Assessment Scale for Children; CRS-R, Conners’ Rating Scales Revised; PDDBI, Pervasive Developmental Disorder Behavior Inventory; CSHQ, Children’s Sleep Habits Questionnaire; PSI, Parenting Stress Index; EA, electroacupuncture; SPECT, single-photon emission computed tomography; TEAS, transcutaneous electrical acupoint stimulation; EIA, enzyme immunoassay; ADQ, Social Adaptive Development Quotient Scale; AVP, arginine-vasopressin; OXT, oxytocin; SEA, sham electroacupuncture; PEDI, Pediatric Evaluation of Disability Inventory; Leiter-R, Leiter International Performance Scale-Revised; CGI-I, Clinical Global Impression-Improvement; RFRLS, Ritvo-Freeman Real Life Scale; RDLS, Reynell Developmental Language Scale; TA, tongue acupuncture; SA, sham acupuncture; GMDS, Griffiths Mental Developmental Scale; SPT, Symbolic Play Test; qEEG, quantitative electroencephalography.

### 2.1. Study subjects

Sufficient sample size can test the feasibility of clinical application of treatment method (47). As shown in Table 1, only one clinical trial had more than 100 participants, which may explain why there is not enough evidence to prove whether acupuncture is effective in the treatment of ASD in the past 15 years (6). The small number of participants in other studies will increase the risk of failing to show the significant treatment benefits of acupuncture (47). Therefore, it is very important to determine the sample size required to test the hypothesis through the effect size before the beginning of the study (48).

Another point that needs to be discussed is that three clinical studies did not mention the exclusion criteria of patients (9, 44, 45). It is worth mentioning that ASD patients are usually accompanied by other diseases, such as neurological disorders, mental disorders, and gastrointestinal disorders (17, 42, 49). In TCM, neurological and mental disorders usually have the same syndrome, so the treatment is roughly the same. This concept is called “*Yi Bing Tong Zhi*” (异病同治, same treatment for the same syndrome with different diseases) (50). However, modern medicine believes that the pathogenesis of these diseases is different, and the treatment methods should also be different. In fact, if the inclusion criteria are narrow, the homogeneity of the study subjects’ characteristics is increased, but the generalizability of the results is reduced. Conversely, broader inclusion criteria may affect researchers’ evaluation of the efficacy of treatment method (51). Therefore, if the core target of the study is to confirm the efficacy of acupuncture in the treatment of ASD, unified and strict inclusion and exclusion criteria will reduce the risk of reporting bias in the clinical trials of acupuncture and make the results more reliable (52).

By further analyzing the clinical study reports in Table 1, we found that the age range of patients included in each clinical trial was different. For example, the maximum age of patients included in the study by Wong et al. (42) was 18 years, while the age of participants in the study by Chen et al. (46) was 11 years. Understandably, this depends on the actual situation of the patients included in the study. However, the golden treatment time for ASD is known to be between the ages of 2 and 6 years (6). Whether adolescent patients will have heterogeneity of ASD due to exposure to more external environmental factors remains to be further discussed (53).

### 2.2. Group setting

The group setting of enrolled patients is an important link in the clinical trial design, because whether the grouping follows the principles of randomization and blinding may have a direct impact on the study results (52). Of the 11 studies included in this review, 6 clinical trials adopted the randomized grouping principle, including single-and double-blind study (6, 38, 42–45). Among them, the participants in five clinical trials were small. So, compared with simple randomization, the restricted randomization may greatly reduce the imbalance between the experimental group and the control group, but the power to test a hypothesis are modest. Because the efficacy evaluation of mental disorders is often based on questionnaires, which inevitably involves the subjective results from doctors, patients, and their families. Therefore, the studies without randomization and blinding design should pay attention to the bias caused by these factors.

The setting of the control group can help researchers judge whether the intervention method applied in the experimental group are superior or non-inferior to the current treatment method in clinic, and assess whether the improvement of patients is attributable to the trial intervention or just a natural progression of the disease over time (51). In all the collected clinical study reports, four studies set up a control group (38, 41, 44, 45). However, it is worth noting that in the study by Surapaty et al. (38), the patients in the experimental group received sensory-occupational integrated therapy at the same time as the control group before receiving LA. The patients in the experimental group in the study by Zhang et al. (41) and Allam et al. (45) also received rehabilitation training and language therapy before acupuncture, respectively. Although their findings ultimately showed that ASD patients receiving additional acupuncture could achieve a greater degree of improvement, they did not strongly explain that acupuncture is an effective treatment for ASD. Nevertheless, Surapaty et al. (38) explored the feasibility of laser acupuncture in the treatment of ASD, while Zhang et al. (41) objectively explored the potential mechanism of acupuncture by measuring the levels of arginine-vasopressin (AVP) and oxytocin (OXT) before and after TEAS. The latter study well exemplifies the need to employ functional measures to substantiate through which mechanism acupuncture, if proven beneficial, may exert its effects.

In all studies grouped by parallel design, we observed that two studies set up sham acupuncture (SA) group. Wong et al. (42, 43) not only reported significant improvement in ASD patients receiving acupuncture, but also mentioned that SA improved ASD-related symptoms to a certain extent. However, these results were only based on the comparison between patients before and after treatment and between acupuncture and SA, not under the comparison of the control group. For SA, some researchers believed that although it mimics the procedure of acupuncture, it did not elicit the therapeutic effect (54). Interestingly, other researchers found that SA can cause physiological reactions similar to acupuncture (55, 56). For example, in a phase 3 randomized clinical trial of acupuncture in the treatment of xerostomia induced by chemo- and radiotherapy for head-and-neck cancer, the researchers found that the SA alleviated patients’ xerostomia, and there was a significant difference in the treatment results between the SA group and the control group (57). In the above-mentioned two clinical studies, the researchers did not set up the control group, so it is impossible to analyze SA at a deeper level. However, the effect of acupuncture can be affected by the psychological state of patients, the operation level of acupuncturists, and doctor–patient relationship (58). Obviously, the final conclusion needs to be clarified by a more scientific study design. In addition, we also observed that Chen et al. (46) assigned different acupoints’ combination in the two groups of patients. Although only two patients were included in this study, the advantage of this grouping method is that it is expected to screen acupoints with better therapeutic effect on ASD, which is worth learning from.

### 2.3. Intervention modality

As mentioned earlier, there are many CAM therapies for ASD, and at least 50% of patients have tried these therapies to varying degrees (10, 11). Therefore, if the purpose of the researcher is to investigate the precise efficacy of acupuncture on ASD, the treatment received by ASD patients before acupuncture is an important factor that cannot be ignored. In other words, researchers should pay attention to maintain the homogeneity of the characteristics of the enrolled patients and avoid the impact of the difference of prior therapy history between patients on the study results (51, 52). In all collected clinical trials, we found that five reports did not mention whether patients received conventional anti-ASD treatment or CAM therapy before acupuncture (9, 40, 43–45). In studies that gave a clear statement, Wong et al. (42) confirmed that the enrolled patients had not taken anti-epileptic drugs or received acupuncture within 6 months. Warren et al. (39) excluded ASD children who had received pharmacological and/or non-pharmacological therapy within 4 weeks. It is worth noting that the duration of efficacy produced by different treatment methods is different. For example, some researchers have found that acupuncture can improve salivary gland dysfunction for up to 1 year (57, 59, 60). Therefore, the ideal characteristics of the enrolled patients should be that they were initially diagnosed and did not receive any treatment, or did not experience any anti-ASD treatment for at least 1 year. Although this view may be slightly impractical in clinical work, researchers should at least explain in the report that the broader inclusion criteria are focused on the generalizability of acupuncture in the treatment of ASD.

In analyzing these clinical trials, we found that patients in the experimental group of four studies received two intervention modalities. In the study by Surapaty et al. (38), the patients in the experimental group experienced sensory-occupational integrative therapy with the patients of control group before receiving acupuncture, and their speech ability and social interaction were finally found to be more improved than those in the control group. Lee et al. (6) found that herbal medicine combined with acupuncture is safer and more effective in the treatment of ASD than placebo combined with acupuncture. Zhang et al. (41) implemented TEAS in ASD patients who received rehabilitation training and found that TEAS was more effective for autistic children with a passive and aloof social interaction style than simple rehabilitation training. Allam et al. (45) implemented acupuncture in patients receiving language therapy and found that the cognitive and language expression abilities of patients were more improved than those receiving language therapy alone. Generally, in order to accurately evaluate the clinical efficacy of a treatment method, patients assigned to the experimental group will only receive investigational treatment. In view of the literature analysis, we found that some researchers believed if patients are preferentially allocated to receive the standard of care rather than an investigational treatment, then the results will tend to show better outcomes in the experimental group than in the control group even if the investigational treatment confers no benefit (51). Therefore, when there is no sufficient evidence to support the effectiveness of acupuncture on ASD, it is still the primary consideration to apply acupuncture alone for the experimental group under the condition of selectivity.

In all collected clinical trials, 6 studies used manual acupuncture and 3 studies used EA. Through in-depth analysis of these study reports, it can be found that there were significant differences in time, frequency, and total sessions of acupuncture. For example, in the study of scalp acupuncture, Yau et al. (9) provided 1 h of treatment to patients, while Allam et al. (45) provided 20 min of treatment to patients. In terms of frequency, Chan et al. (44) provided 5 treatment sessions a week for 6 weeks, while Chen et al. (46) provided 3 treatment sessions a week for 8 weeks. In addition, in the study conducted by Zhao et al. (40), patients received EA for a total course of 12 to 24 months. There could be several possible reasons to explain this issue. Initially, we found that almost all researchers did not declare that they followed Standards for Reporting Interventions in Clinical Trials of Acupuncture (STRICTA) (61). Moreover, there were significant differences between the study design of clinical trials, and the TCM theoretical knowledge and practical experience of acupuncturists (24, 51, 52). Eventually, different study objectives can lead to differences in the application time and treatment course of acupuncture in some studies. For example, Warren et al. (39) aimed to determine whether ASD patients can tolerate the intervention of acupressure combined with acupuncture, so they introduced acupuncture at mid-treatment of acupressure only. Zhao et al. (40) aimed to evaluate the efficacy of EA in the treatment of ASD by single-photon emission computed tomography, so the patients they included received EA for at least 12 months.

In addition, we also found two other intervention modalities, namely LA and TEAS. LA is one of many acupuncture therapies. Its common characteristic with EA and TEAS is that it depends on the control of equipment. Study showed that compared with manual acupuncture, LA has the advantages of simple application, accurate “dose” measurements, painlessness, and noninvasiveness (62). Surapaty et al. (38) implemented LA three times a week in ASD patients for 30 sessions, and found that it could significantly improve the speech ability and social interaction of ASD patients. TEAS uses self-adhesive electrodes placed on the surface of acupoints, instead of inserting the needle into the skin like a manual acupuncture. Its application follows the meridian and acupoint theory of TCM. By activating nerve endings or fibers to generate action potentials, it transmits signals to the central nervous system, so as to produce specific chemical mediators to induce relevant physiological effects (63–65). The researchers believed that the advantage of this intervention can improve treatment compliance by eliminating the patients’ fear of the needle (66). Zhang et al. (41) found that TEAS can significantly improve the ASD children with passive and aloof social interaction on the basis of rehabilitation training by giving patients TEAS 5 times a week for 60 sessions. Currently, TEAS is also used to treat other diseases, and few adverse effects have been reported (67). However, more studies are needed to confirm whether LA and TEAS are better than manual acupuncture and EA in the treatment of ASD.

### 2.4. Acupoints selection

In TCM theory, acupoints are connected with *Zang-Fu* organs through meridians. Stimulation of acupoints can regulate the physiological functions of *Zang-Fu* organs and correct pathological changes through a series of mechanisms, so as to relieve symptoms and signs (68, 69). There are more than 400 named TCM acupoints on the human body (17, 70). Each acupoint is connected to one or more *Zang-Fu* organs by meridians, so the function of each acupoint is different. Therefore, discussing the function of acupoints and the impact of different acupoints’ combination on the study results should be the core content of reviewing acupuncture clinical trials. In this review, although each study selected different acupoint combinations to treat ASD, we can still find some frequently used acupoints. In all collected clinical trials, the utilization rate of SP6 acupoint was 45.5%, and ST36 acupoint was 36.4%, while the utilization rate of GV20, LI4, HT7, EX-HN1 and EX-HN3 acupoints was 27.3%. Among them, GV20, EX-HN1, and EX-HN3 are the scalp acupoints with the highest frequency of use (Table 1). In addition, Yau et al. (9) believed that EX-NH3 represented *Sishencong* acupoint, while Chen et al. (46) believed that EX-NH3 represented *Yintang* acupoint. In fact, according to the international code of human acupoints, the corresponding code of *Sishencong* acupoint is EX-HN1, while that of *Yintang* acupoint is EX-HN3 (71, 72).

In the meridian theory, SP6 (*Sanyinjiao*) is an acupoint on the spleen meridian. Because it is located at the intersection of the Jueyin Liver Channel of Foot (足厥阴肝经), the Taiyin Spleen Channel of Foot (足太阴脾经), and the Shaoyin Kidney Channel of Foot (足少阴肾经), it is considered to have the effect of nourishing the liver and kidney and strengthening the spleen and stomach in TCM (73). ST36, also known as *Zusanli*, belongs to Yangming Stomach Channel of Foot (足阳明胃经), which has the function of regulating the spleen and stomach and enhancing the immunity of the body (74). LI4 (*Hegu*) belongs to the Large Intestine Channel of Hand-Yangming (手阳明大肠经). Its function mainly includes regulating *Qi-Blood* and refreshing the brain and opening the orifices (75). HT7 is the international code of *Shenmen*, which is the acupoint on the Shaoyin Heart Channel of Hand (手少阴心经). In TCM theory, it has the function of promoting digestion, helping sleep and regulating visceral nerves (76, 77). Based on TCM theory, the pathogenesis of ASD is related to the abnormal function of the heart, liver, spleen, and kidney. This may explain why these four acupoints are used more frequently in the study of acupuncture in the treatment of ASD. GV20 (*Baihui*), EX-HN1 (*Sishencong*) and EX-HN3 (*Yintang*) are acupoints located on the head, of which the former belongs to the Governor Vessel (督脉) and the latter two belong to the extra-meridian points (经外奇穴) (70). In TCM theory, the functions of these acupoints are mainly related to awakening the brain and calming the mind. Their clinical application mainly involves the treatment of neurological or mental diseases, such as dementia, stroke, insomnia, epilepsy, forgetfulness, depression, and ASD (42, 78, 79).

In TCM theory, the syndromes of ASD can be summarized into two categories: (1) congenital disorders of multiple organs with a dominant insufficiency of kidney-*jing* and (2) disorders caused by heart-*qi* deficiency. Among them, the former was observed to be mainly physical and intellectual disability, manifested as inability to sit at the age of 6 months, inability to stand at the age of 15 to 18 months, ataxia, and delayed speech development. The latter is mainly delayed echolalia, including poor communication ability and repetitive and stereotyped behaviors (26). According to TCM theory, the brain is connected to the heart, liver, spleen, and kidney through meridians. Acupuncture at acupoints related to the brain and *Zang-Fu* organs can regulate the *Qi-Blood*, so as to regulate the development and function of *Zang-Fu* organs (26, 80). The brain and *Zang-Fu* organs are connected with each other by multiple meridians. This may explain why different researchers choose different acupoints to treat ASD (81).

From the perspective of modern medicine, ASD can be divided into dysfunction in the neural structure and biochemical abnormalities of the brain tissue (38). Although the pathogenesis of ASD is still unclear, from an anatomical point of view, the symptoms of ASD are closely related to the functional abnormalities of the telencephalon, cerebellum, and diencephalon, which are responsible for managing memory, learning, emotion, sleep, sensation, movement, language, and psychology (82). Some neurotransmitters and neurotrophins, such as arginine-vasopressin (AVP), oxytocin (OXT), glutamic acid, γ-aminobutyric acid, endorphin, serotonin, cannabinoid, dopamine, norepinephrine, and brain-derived neurotrophic factor (BDNF) are considered to be involved in the pathogenesis of ASD and are often used as objective indicators for researchers to evaluate the clinical efficacy of treatment methods (17, 51, 83, 84).

Previous studies have shown that EA at SP6 can induce increased functional connectivity between amygdala, brainstem, and hippocampus, and can simultaneously act on presynaptic dopamine transporter and postsynaptic dopamine receptors (73, 85). In another study, the researchers used manual acupuncture to stimulate ST3, ST6, LI4, ST36, and SP6 acupoints in patients with salivary gland dysfunction and found that the levels of perivascular peptides (calcitonin gene-related peptide, neuropeptide Y, and vasoactive intestinal polypeptide) in saliva were significantly increased (86). In addition, Wattanathorn et al. (87) found that laser acupuncture at the HT7 can stimulate the dopaminergic system, free radicals, and the cholinergic system through acetylcholinesterase and monoamine oxidase-B to induce an increase in acetylcholine and dopamine. In the studies included in this review, Zhang et al. (41) found that the plasma AVP level in ASD patients was significantly higher than that in the control group by using TEAS to stimulate the combination of acupoints including LI4, PC6, ST36, and SP6. Zhao et al. (40) stimulated LI4, LI11, ST36, and SP6 acupoints in ASD patients with EA and found that the intracerebral multiple focal radioactivity distribution defect areas were partially filled. These evidences fully connect acupoint function with neurotransmitters, and undoubtedly build an exploratory bridge between TCM and modern medicine.

Studies have confirmed that stimulation of GV20 acupoint can regulate the expression of BDNF in the brain, so as to improve memory and emotion by restoring synaptic plasticity (88). In addition, stimulation of EX-HN1 acupoint with EA can increase the concentration of monoamine in human body, so as to play an antidepressant role (89). A study on Alzheimer’s disease showed that EA at GV20 and EX-HN3 acupoints could effectively improve the learning and memory of mice and reduce the expression of interleukin-1β (IL-1 β), interleukin-6 (IL6), and tumor necrosis factor-α (TNF-α) (90). Yau et al. (9) implemented manual acupuncture in ASD patients by selecting a combination of acupoints containing GV20 and EX-HN3, and found that 97% of patients had significant improvement of verbal communication problems. Wong et al. (42) found that patients’ language comprehension and self-care ability were significantly improved by using EA to stimulate acupoints such as EX-HN1, EX-HN3, HT7, and SP6. However, Chen et al. (46) selected the combination of EX-HN1, EX-HN3, PC6, AT3, ST36, and LR3, as well as EX-HN1, EX-HN3, HT7, shounaodian, KI3, and SP6 acupoints to perform EA in ASD patients, and found that movement assessment battery for children (MABC2), pediatric functional independence measure (WeeFIM), and clinical global impression-improvement (CGI-I) scores were not satisfactory.

Based on the above analysis, it can be found that all researchers used the combination of multiple acupoints to perform acupuncture for ASD patients. Therefore, it is difficult to analyze the specific efficacy and potential mechanism of a single acupoint in the treatment of ASD. However, the comparative analysis of these acupoints’ combination may help to screen out the acupoints with high utilization rate or the optimal application effect, so as to further combine them for high-quality research and lay a foundation for establishing the “gold standard” of acupoint selection for acupuncture in ASD. In addition, in the future study, a problem that needs researchers’ attention is that acupuncture is carried out in clinical practice under the guidance of TCM theory. Therefore, the relevant TCM background and meridian knowledge should be an indispensable part of every acupuncture clinical study report.

### 2.5. Outcome evaluation and safety

Since acupuncture has not been approved as the standard CAM therapy for ASD, it is very important to evaluate the study results scientifically, comprehensively and effectively for the accumulation of clinical evidence. Based on the characteristics of ASD, the scale is used as the main method for diagnosis and efficacy evaluation. From the information provided in Table 1, it can be found that nine studies used scales to evaluate the efficacy of acupuncture in ASD patients, of which six used more than three scales (6, 39, 41–43, 46). The use of multiple scales is conducive to the comprehensive evaluation of the research results, especially when the improvement effect of placebo or SA is also observed, the improvement effect of acupuncture on ASD can be found from the comparison. For example, in the study conducted by Wong et al. (43), patients who received acupuncture and SA showed significant improvement in Griffiths Mental Developmental Scale (GMDS; developmental index), Ritvo-Freeman Real Life Scale (RFRLS; behavioral index), and Reynell Developmental Language Scale (RLDS, language index), while patients who received acupuncture had more significant improvement in the total score and functional quotient of the Functional Independence Measure for Children (WeeFIM; functional ability index) than patients who received SA. In addition, considering the specific patients with ASD, feedback from parents is also an important evaluation index that cannot be ignored. In all collected clinical trials, 6 studies used parental reports to evaluate the clinical efficacy of acupuncture (6, 38, 39, 41, 42, 46). Although these scales can effectively integrate information from patients, parents, and doctors, they are still not enough to support the effectiveness of acupuncture in the treatment of ASD, because there is still a lack of objective evidence.

In all studies using the scale to evaluate the outcomes, we found that only Zhang et al. (41) measured the levels of plasma AVP and OXT in patients after TEAS, which may help to explore the potential mechanism of acupuncture in the treatment of ASD, especially related acupoints, from the perspective of modern medicine. In addition, we also found that Zhao et al. (40) applied SPECT to evaluate the effectiveness of acupuncture in ASD, and Chan et al. (44) used quantitative electroencephalography (qEEG) to measure the neurophysiological changes of patients to evaluate the efficacy of acupuncture in ASD. Although these two studies also accumulated objective clinical evidence for acupuncture in the treatment of ASD, they did not comprehensively evaluate the results using scales or other methods. We speculated that this may be related to the main objectives of the researchers, that is, to confirm whether SPECT or qEEG can be used as a tool to evaluate the efficacy of acupuncture.

In the following analysis, we found that most studies only evaluated the outcomes at the end of treatment, and only Lee et al. (6) and Warren et al. (39) conducted additional evaluation in the midterm of treatment, which were at four time points: baseline and at 6, 12, and 24 weeks after the beginning of treatment, and at three time points: pre-, mid-, and post-intervention, respectively. Multiple evaluation of the patient’s state at different time points may be helpful to accurately judge the effectiveness of acupuncture. One problem that needs to be paid attention to is the compliance of the patients and their parents, because it does not rule out that the patients’ parents may seek other CAM therapies during acupuncture and interfere with the study results (11, 12). In addition, another problem worth discussing is that we found that all studies focused on the short-term efficacy of acupuncture without follow-up. It has been found that the improvement effect of acupuncture on lower limb motor dysfunction after stroke can be maintained for 2 months, while the improvement effect on depression and anxiety caused by chronic insomnia can be maintained for 3 months (91, 92). Therefore, for accumulating evidence of the effectiveness of acupuncture in the treatment of ASD, follow-up data are an important content that cannot be ignored.

For the safety of acupuncture in the treatment of ASD, none of the studies included in this review reported serious adverse effects. Although Wong et al. (42) observed that a few patients had mild adverse effects of superficial bleeding or irritability during acupuncture, this may be related to the manipulation of acupuncturists. In addition, although Zhang et al. (41) reported that TEAS was safe in the treatment of ASD, it caused gastrointestinal toxicity in other study (93). Therefore, considering that the sample size of these clinical trials is very small, it is important to carry out large cohort studies to confirm the safety of acupuncture in the future.

## 3. Future prospects

Based on the above comprehensive analysis, though a potentially beneficial treatment for ASD, acupuncture cannot be recommended at this time in clinical practice, due to the limited and low-quality evidence currently available. Indeed, the scarcity and poor quality of clinical trials are the existing problems in this research field, which may also be the main reason why the effectiveness of acupuncture is difficult to be recognized and regarded as the grade C treatment for ASD (25). We searched the clinical trial databases in China^1^ and the United States^2^ and found that there were 12 registered studies, of which 2 were ongoing studies (Table 2). Based on the above viewpoints, we believe that the potential value of acupuncture in the treatment of ASD still needs to be discovered through more high-quality studies. Therefore, in the future study, researchers should scientifically and rigorously design the experimental links such as study subjects, group setting, intervention modality, acupoint selection, and outcome evaluation, so as to improve the credibility of the results by carrying out high-quality research. In this process, it is essential to follow the guidance of STRAICTA and Consolidated Standards of Reporting Trials (CONSORT), because it is very important for readers to evaluate the effectiveness of the trial and the replication of successful discovery (94–96).

**Table 2:** Registered clinical trials on acupuncture in the treatment of ASD in China and United States.

Trial name/number/status	Study design	Patient population and interventions	Outcome measures	Study execute time	Country
Visualization of diffusion tensor imaging in acupuncture treatment of children with autism/ChiCTR2100047559/recruiting	Randomized Control Trial (parallel)	Control group:18 (SA) Experimental group:18 (MA)	CARS, ABC^1^, AETC, Diffusion tensor imaging of nerve fiber bundles, Neuroelectrophysiological test	From June/2021 to June/2023	China
Positive Regulation of the Social and Emotional Disorders and Brain-Gut Axis Mechanisms for Children with Autism Spectrum Disorders by Jin’s 3 needle Technique/ChiCTR2000029357/not yet recruiting	Randomized Control Trial (parallel)	Jin’3 needle technique treatment group A: 138 (excluding gastrointestinal 3-needle) Jin’3 needle technique treatment group B: 138 (including gastrointestinal 3-needle) Control: 138 (conventional rehabilitation training)	ADOS, Conners’ questionnaire, CARS, ABC^1^, BSID, WISC, Wexner score	From January/2020 to December/2023	China
The efficacy of scalp acupuncture combined with TEACCH in rehabilitation of autism spectrum disorder/ChiCTR1900023247/not yet recruiting	Quasi-randomized controlled	Scalp acupuncture treatment group: 50 TEACCH group: 50	PEP-3 Autism Developmental Assessment Scale	From January/2018 to December/2019	China
Methodological study on the evaluation of clinical characteristics and efficacy of acupuncture in the treatment of autism spectrum disorders in Chinese medicine/ChiCTR2200056901/completed	Cohort study	Experimental group:1658 (Acupuncture combined with Conventional rehabilitation training)	CARS, Gesell Development Diagnosis Scale, ABC^1^, PEP-3 Autism Developmental Assessment Scale	From June/2020 to May/2022	China
Effect of transcutaneous vagus nerve stimulation at auricular concha on patients with autism spectrum disorder: a random-controlled trail study/ChiCTR-INR-17012642/recruiting	Randomized Control Trial (parallel)	Experimental group:20 (EA) Control group: not mentioned	Cytokines, fMRI, AETC, CARS, The Screen for Child Anxiety Related Emotional Disorders, Child Quality of Life, Neurotransmitters, Social Responsiveness Scale, Empathizing/Systemizing quotient, child version, WISC, Conners’ questionnaire, behavior teat	From March/2017 to March/2020	China
Effects of acupuncture on the brain neurochemical substrate metabolism status in non-verbal autistic children/ChiCTR-IPR-17010558/recruiting	Randomized Control Trial (parallel)	Control group: 20 (N/A) Experimental group: 20 (MA)	ABC^1^, RDLS, Gesell Development Diagnosis Scale or WISC-IV, MRS	From February/2017 to December/2018	China
The brain functional promotion of auricular concha electro-acupuncture on children with autism spectrum disorder/ChiCTR-IOR-16010252/recruiting	Randomized Control Trial	Experimental group:100 (EA) Control group: not mentioned	CARS, psycho-educational profile, fMRI	From January/2017 to December/2019	China
Investigating the Use of Acupressure and Acupuncture With Children With Autism II/NCT00935701/completed	Single-arm trial without control	Experimental group:10 (Acupressure and MA)	Conners questionnaire, Change in Children’s Sleep Habits Questionnaire, Change in Parenting Stress Index	From July/2009 to February/2011	United States
Randomized Control Trial of Using Acupuncture In Children With Autistic Spectrum Disorder/NCT00352352/completed	Randomized Control Trial (parallel)	Experimental group:15 (TA) Control group: 15 (not mentioned)	RFRLS, WeeFIM, PSI, CGIS	Not provided	Hong Kong, China
Randomized Controlled Trial of Acupuncture Versus Sham Acupuncture in Autistic Spectrum Disorder/NCT00352248/completed	Randomized Control Trial (parallel)	Experimental group:25 (TA) Control group: 25 (not mentioned)	GMDS, RFRLS, RLDS, SPT, WeeFIM	Not provided	Hong Kong, China
Randomized Control Trial of Using Tongue Acupuncture in Autistic Spectrum Disorder Using PET Scan for Clinical Correlation/NCT00355329/completed	Randomized Control Trial (parallel)	Experimental group:15 (TA) Control group: 15 (not mentioned)	ATEC, RLDS, SPT, WeeFIM, CGIS, PET	Not provided	Hong Kong, China
Use of Acupuncture in Autistic Spectrum Disorder/NCT00346736/completed	Randomized Control Trial (Crossover Assignment)	Acupuncture group: 40 (EA) Sham acupuncture group: 40 (EA)	ABC1, RFRLS, WeeFIM, PEDI, RLDS, CGIS, SPT, Leiter International Performance Scale-Revised	From May/2005 to June/2006	Hong Kong, China

SA, sham acupuncture; MA, manual acupuncture; CARS, Childhood Autism Rating Scale; ABC^1^, Autism Behavior Checklist; AETC, Autism Efficacy Assessment Scale; ADOS, Autism Diagnostic Observation Schedule; BSID, Bayley Scales of Infant Development; WISC, Wechsler Intelligence Scale for Children; TEACCH, treatment and education for autistic and related communication handicapped children; RDLS, Reynell Developmental Language Scale; MRS, magnetic resonance spectrum; EA, electroacupuncture; fMRI, functional magnetic resonance imaging; TA, tongue acupuncture; RFRLS, Ritvo-Freeman Real Life Scale; WeeFIM, functional independence measure for children; PSI, Parental Stress Index; CGIS, Clinical Global Impression Scale; GMDS, Griffiths Mental Developmental Scale; SPT, symbolic play test; ATEC, Autism Treatment Evaluation Checklist; PET, 18F-fluorodeoxyglucose positron emission tomography; PEDI, Pediatric Evaluation of Disability Inventory.

In this review, although all collected clinical trials reported that the acupoints they selected were suitable for acupuncture in the treatment of ASD, at present, no single acupuncture technique or set of acupoints is recognized as the “gold standard” for the treatment of ASD. Therefore, researchers should first make a perfect group setting on the basis of large cohort or multicenter research. In addition, researchers should also pay attention to the appropriate randomization of controls receiving SA, as well as the different acupoints should be added to the experimental group to screen out a better acupoints’ combination (54). Secondly, scientific outcome evaluation should not only include a variety of scales, but also consider the measurement of objective indicators, such as neurotransmitters and neurotrophins, because it may help to clarify the potential mechanism of acupuncture in the treatment of ASD (47, 51). Finally, regular follow-up of patients after treatment to confirm the long-term efficacy of acupuncture can add more convincing evidence to the study results (51, 52).

At present, the research on the potential mechanism of acupuncture in the treatment of ASD mainly focuses on using modern medical technology to explore the correlation between acupoints and neurotransmitters, neurotrophins, or inflammatory factors (41, 75, 78, 83). However, due to the complexity of meridian theory and the diversity of acupoint selection, as well as the unclear pathogenesis of ASD, the evidence obtained from the existing functional experiments showed that it is still difficult to support the view that acupuncture can effectively treat ASD (11, 13, 36). Since TCM and modern medicine are two different medical theoretical systems, how to combine TCM theory with modern medical experimental methods is the main problem that researchers need to pay attention. Recently, significant breakthroughs have been made in the visualization of human meridians, and the research on identifying syndrome biomarkers from the perspective of TCM using omics technologies is also increasing (97–99). These clues may help researchers to unravel the mystery of acupuncture in the future.

## 4. Conclusion

To sum up, we comprehensively analyzed and discussed the current clinical study reports on acupuncture in the treatment of ASD in terms of study subjects, group setting, intervention modalities, acupoint selection, outcome evaluation, and safety. We found that the studies were not comparable due to differences in the study design, and the existing findings were insufficient to support the effectiveness of acupuncture in treating ASD due to the lack of high-quality research. However, these studies may lay the foundation for reliably assessing the potential efficacy and safety of acupuncture in ASD, which are worthy of high-quality research in the future. Therefore, we believed that following the guidance of STRICTA and CONSORT, screening the optimal combination of acupoints applying a rigorous scientific study design, and performing the related functional experiments may be the effective way to convincingly test the hypothesis that acupuncture may be beneficial in ASD patients. The significance of this review is to integrate and improve the TCM background knowledge of acupuncture in the treatment of ASD, and put forward comprehensive opinions on the study design of clinical trial combined with modern medical theory, so as to provide reference for the development of high-quality research in the future.

## Author contributions

S-QZ, XL, and J-CL: conceptualization. S-QZ: methodology, writing – review and editing, and project administration. XL, J-CL, Q-QL, FZ, and S-QZ: investigation. XL and S-QZ: writing – original draft preparation. J-CL: supervision. All authors contributed to the article and approved the submitted version.

## Funding

This study was supported by grants from the Shaoguan Science and Technology Plan Projects in 2020, China (grant no. 200812094530421).

## Conflict of interest

The authors declare that the research was conducted in the absence of any commercial or financial relationships that could be construed as a potential conflict of interest.

## Publisher’s note

All claims expressed in this article are solely those of the authors and do not necessarily represent those of their affiliated organizations, or those of the publisher, the editors and the reviewers. Any product that may be evaluated in this article, or claim that may be made by its manufacturer, is not guaranteed or endorsed by the publisher.

## Footnotes

^1^http://www.chictr.org.cn/index.aspx

^2^https://clinicaltrials.gov/ct2/home
